# Behavioral Sleep Therapies During the Perinatal Period: A Scoping Review

**DOI:** 10.1007/s40675-026-00359-x

**Published:** 2026-03-03

**Authors:** Jennifer N. Felder, Bernadette McClelland, Candance Sorensen, Meghan Brown, Richelle Mah

**Affiliations:** 1 Osher Center for Integrative Health, University of California, San Francisco, CA USA; 2 Department of Psychiatry and Behavioral Sciences, University of California, San Francisco, CA USA

**Keywords:** Cognitive behavioral therapy for insomnia, Mindfulness-based interventions, Perinatal insomnia, Behavioral sleep therapies

## Abstract

**Purpose of Review:**

Perinatal insomnia is common and consequential. This review presents findings from randomized controlled trials of behavioral sleep therapies in perinatal samples published since 2020.

**Recent Findings:**

Eleven studies were included; seven investigated cognitive behavioral therapy for insomnia (CBT-I) and four investigated mindfulness-based interventions (MBI). CBT-I is effective for improving perinatal sleep. Benefits attenuate in the early postpartum period, and re-emerge around six months postpartum. Impact on mental health outcomes is mixed, possibly due to variations in comparator groups and assessment timepoints. Two of the MBI trials were adequately powered to investigate effects on insomnia severity, and suggest that MBIs may be effective for reducing insomnia during pregnancy.

**Summary:**

Future work should evaluate the effectiveness of behavioral sleep therapies in real world settings, examine benefits beyond six months postpartum, evaluate impacts on offspring, conduct rigorous research to better quantify mental health benefits, and elucidate for whom MBI is indicated vs. CBT-I.

## Introduction

In the past decade, accumulating evidence has reshaped our understanding of the clinical significance of insomnia during pregnancy and the postpartum period. More than one-third of pregnant individuals experience clinically significant insomnia symptoms [1], defined by difficulty initiating or maintaining sleep with accompanying distress, impairment, and daytime symptoms [2]. Data characterizing onset timing of insomnia symptoms in pregnant samples are scarce; in one study of 106 pregnant women with elevated insomnia symptoms, approximately 40% reported their symptoms began during pregnancy [3]. Importantly, insomnia is not limited to the third trimester, when physical discomfort and birth-related worries peak; [4] it is also highly prevalent in the first and second trimesters [1]. Further, perinatal insomnia often persists across the transition to parenthood. In a longitudinal, population-based study in Norway, among those with insomnia disorder at 32 weeks’ gestation, 68% continued to experience insomnia at 8 weeks postpartum and 50% at 2 years postpartum [5]. Without intervention, perinatal insomnia may be unlikely to remit spontaneously.

Although perinatal sleep disturbance is normative, perinatal insomnia specifically is not a benign condition. It is associated with increased risk of maternal depression and preterm birth [6–9]. Emerging evidence suggests that perinatal insomnia could have intergenerational impacts, ranging from effects on fetal brain development (potentially via fetal programming of disease) [10, 11], toddler cognition [12], risk for ADHD [13], and poorer social-emotional development from infancy through early childhood (potentially via impacts on parenting behaviors) [14–16]. Taken together, this evidence has catalyzed a growing focus on the development and evaluation of perinatal insomnia interventions.

Behavioral sleep therapies may be particularly well-suited for perinatal individuals, who typically prefer non-pharmacological approaches for insomnia [17]. Until recently, it was uncertain whether standard behavioral sleep therapies would translate effectively to perinatal populations, whose sleep is disrupted by physiologic factors and caregiving demands that are not easily modified (e.g., nocturia, physical discomfort, nighttime infant care) [4, 18]. As the majority of empirical studies evaluating behavioral sleep therapies for perinatal populations have been published within the last five years, a scoping review is well-timed to integrate these emerging findings and identify remaining gaps.

### Search Parameters

**Fig. 1:**
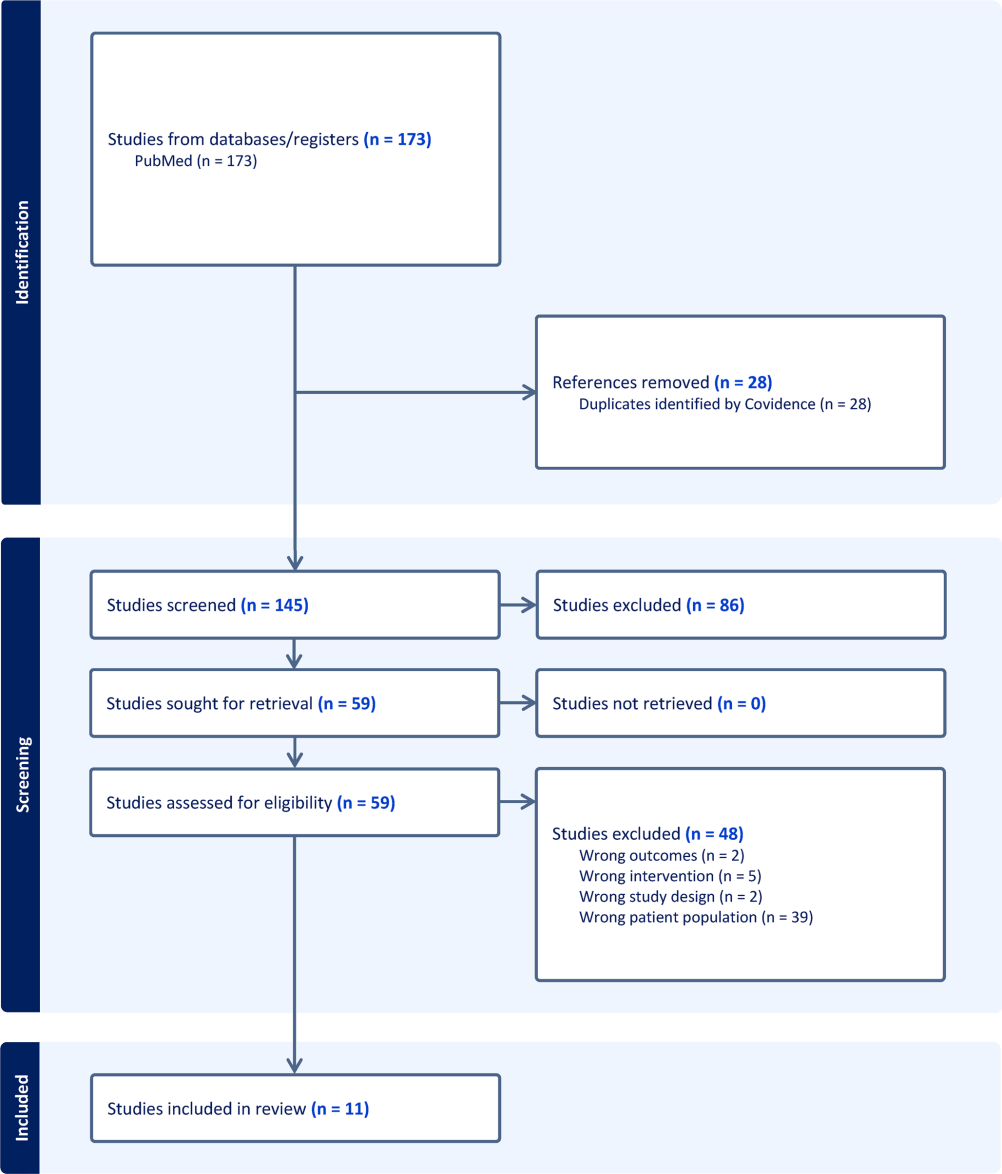
PRISMA Flow Diagram

This scoping review included randomized controlled trials evaluating behavioral sleep therapies among pregnant or postpartum individuals. Eligible studies were randomized controlled trials of behavioral or cognitive behavioral sleep interventions, published in English on or after January 1, 2020, that enrolled pregnant and/or postpartum individuals selected for sleep complaints (e.g., elevated insomnia severity, poor sleep quality), and specified a sleep-related primary outcome. There were no restrictions on geographic location or study setting. We excluded trials that evaluated stand-alone educational interventions (e.g., sleep hygiene), lifestyle approaches (e.g., exercise), environmental modifications (e.g. eye masks, ear plugs), or complimentary practices (e.g., music, aromatherapy). These approaches may support sleep but do not target behavioral or cognitive mechanisms. We searched PubMed on October 2, 2025 using terms related to pregnancy, postpartum, insomnia or sleep, and behavioral interventions, then imported results into Covidence. Covidence facilitated automated duplicate removal, independent title and abstract screening, full-text review, and resolution of reviewer conflicts. See Fig. 1.

### Cognitive Behavioral Therapy for Insomnia

Cognitive Behavioral Therapy for Insomnia (CBT-I) [19, 20] has been widely demonstrated as an effective intervention for treating insomnia in non-pregnant populations; [21, 22] accordingly, it is recommended by the American College of Physicians as the first-line treatment for insomnia [23]. CBT-I targets the maladaptive behaviors and unhelpful cognitions that perpetuate sleep disturbances. It includes five treatment components: sleep hygiene education, stimulus control to associate the bed with sleep, time in bed restriction to consolidate sleep, relaxation, and cognitive therapy to counter unhelpful beliefs about sleep.

Table 1 provides an overview of findings from CBT-I trials in perinatal populations, including the study population, intervention, comparator(s), primary outcome, timepoints, and results. The majority of studies covered by this review used the Insomnia Severity Index (ISI [24]) to measure insomnia severity and the Pittsburgh Sleep Quality Index (PSQI) to measure sleep quality [25]. The ISI is a 7-item self-report measure assessing severity of sleep latency and sleep maintenance problems, sleep dissatisfaction, associated impairment, distress, and noticeability of symptoms. Total scores range from 0 to 21; scores 0–7 indicate no clinically significant insomnia, 8–14 indicate subthreshold insomnia, 15–21 indicate clinical insomnia of moderate severity, and 22–28 indicate clinical insomnia of severe severity. The PSQI is a 21-item self-report measure assessing sleep duration, sleep disturbance, sleep latency, daytime dysfunction, sleep efficiency, overall sleep quality, and requiring medication to sleep. Total scores range 0–21, and scores > 5 indicate poor sleep quality.

**Table 1:** Summary of findings from CBT-I trials

Study	Population	Intervention	Comparator(s)	Primary outcome	Timepoints	ISI Results	PSQI Results	Depression Results
Felder et al., 2020	Pregnant; Elevated insomnia severity	Digital CBT-I	Standard care	Insomnia severity	Baseline, post intervention (10 weeks post-randomization), follow-up (18 weeks post-randomization)	Greater improvements at post-intervention (-5.9 vs. -2.3; Cohen’s d=-1.03) and follow-up (-5.58 vs. -3.06; Cohen’s d=-0.54) for digital CBT-I vs. standard care participants.	Greater improvements at post-intervention (-3.1 vs. -0.2; Cohen’s d=-1.04) and follow-up (-2.34 vs. − 0.9; Cohen’s d=-0.34) for digital CBT-I vs. standard care participants.	Greater improvements at post-intervention (EPDS total − 2.2 vs. -0.1; Cohen’s d=-0.39) and follow-up (EPDS total − 2.34 vs. 0.05; Cohen’s d=-0.48) for digital CBT-I vs. standard care participants.
Felder et al., 2022	*	*	*	*	Baseline, 3 months postpartum, 6 months postpartum	Change from baseline to 3 and 6 months postpartum did not differ between digital CBT-I and standard care participants.	Change from baseline to 3 and 6 months postpartum did not differ between digital CBT-I and standard care participants.	Greater improvements (EPDS) for digital CBT-I vs. standard care from baseline to 3- (-0.24 vs. +0.03) and 6 months (-0.18 vs. -0.04) postpartum.
Kalmbach et al., 2020	Pregnant; Elevated insomnia severity	Digital CBT-I	Sleep Hygiene Education (SHE)	Insomnia severity	Baseline, 1 week post-intervention, 6 weeks postpartum	Greater improvements at post-intervention among CBT-I (-4.91; Cohen’s d = 0.86) vs. SHE participants (-1.18). Change from baseline to 6 weeks. postpartum did not differ between digital CBT-I and SHE.	Greater improvements at post-intervention among CBT-I (-2.98, Cohen’s d=0.93) vs. SHE participants (0.16). Change from baseline to 6 weeks. postpartum did not differ. between digital CBT-I and SHE.	Change (EPDS) from baseline to 6 weeks postpartum did not differ between groups.
Mackinnon et al., 2025	Pregnant; Elevated insomnia severity	Face-to-face CBT-I, then switched to telehealth	Treatment as usual (TAU)	Insomnia severity	Baseline, 1 week post-intervention, 6 months postpartum	Greater improvements from baseline to post-intervention and 6 months postpartum for CBT-I vs. TAU participants.	Greater improvements from baseline to post-intervention and 6 months postpartum for CBT-I vs. TAU participants.	Not collected.
Manber et al., 2023	Pregnant; Insomnia disorder	Face-to-face CBT-I	Modified pseudo- desensitization therapy for insomnia	Insomnia Severity	Baseline, 8 weeks postpartum, 18 weeks postpartum, 30 weeks postpartum	Lower insomnia severity at 30 weeks postpartum among CBT-I vs. control participants (6.59 vs. 8.70; Cohen’s d = 0.41). No between-group differences at 8 or 18 weeks postpartum.	Not collected.	No between-group differences (EPDS) at any timepoint.
Quin et al., 2024	Pregnant; Elevated insomnia severity; Nulliparous	(1) Telehealth/ email CBT-I (2) Responsive bassinet	Sleep Hygiene Education	Insomnia severity	Baseline, 36 weeks’ gestation, 2 months postpartum, 6 months postpartum, 12 months postpartum	Compared to SHE, average postpartum insomnia severity was lower for CBT-I (-2.01, Cohen’s d = 0.56) but not RB (-0.87, Cohen’s d = 0.25).	Not collected.	Compared to SHE, depression symptoms (PROMIS) were lower for CBT-I (48.76 vs. 51.77) only at 6 months postpartum. No differences between SHE and RB at any timepoint.
Verma et al., 2023	Postpartum; Elevated insomnia severity	(1) Telehealth/ email CBT-I (2) Light-dark therapy	Treatment as usual	Insomnia severity	Baseline, midpoint intervention, post-intervention, 1 month follow-up	Greater improvements at post-intervention in CBT-I vs. TAU (-7.58 vs. -2.73, Cohen’s d=-2.01) and LDT vs. TAU (-6.39 vs. -2.73, Cohen’s d = 1.52). No change in CBT-I or LDT groups from post-intervention to follow-up.	Not collected.	Changes from baseline to post-intervention (PROMIS) did not differ for CBT-I vs. TAU or LDT vs. TAU.

*This paper reports long-term follow-up findings from the trial in the row above

Abbreviations: *CBT-I* Cognitive Behavioral Therapy for Insomnia, *ISI* Insomnia Severity Index, *PSQI* Pittsburgh Sleep Quality Index, *EPDS* Edinburgh Postnatal Depression Scale, *SHE* Sleep Hygiene Education, *TAU* Treatment as Usual, *RB* Responsive Bassinet, *PROMIS* Patient-Reported Outcomes Measurement Information System, *LDT* Light-Dark Therapy

Manber and colleagues were among the first to demonstrate efficacy of CBT-I during pregnancy. While this paper did not meet the inclusion criteria for our review due to a publication date outside the timeframe (2019), we provide a brief summary to aid in interpretation of long-term follow-up data reported in 2023. Pregnant participants with insomnia disorder (*N* = 179) in the United States were randomly assigned to five weekly sessions of either therapist-delivered CBT-I or active control (i.e., modified pseudo-desensitization therapy that included perinatal and infant sleep education, and exercises to desensitize to sleep-related distressing situations) [26]. The CBT-I intervention was modified for the perinatal period, including setting a lower limit for the time-in-bed recommendation at 5.5 h and providing education about perinatal and infant sleep. Relative to control participants, participants randomized to CBT-I had significantly greater improvements in insomnia symptom severity and self-reported total wake time during the sleep period at postintervention. Postpartum results published in 2023 revealed participants randomized to CBT-I had lower insomnia severity at 30-weeks (but not 8- or 18-weeks) postpartum, as well as lower total wake time at 8-, 18-, and 30-weeks postpartum, compared to participants assigned to control [27]. 

Pregnant people need timely access to CBT-I, yet access is often limited by a shortage of trained CBT-I clinicians and long waitlists [28]. Thus, in the last 5 years, much of the research evaluating CBT-I in pregnancy has focused on scalable delivery formats, such as digital apps. Two independent research labs have demonstrated the efficacy of digital CBT-I during pregnancy; both used the Sleepio program. Sleepio’s animated therapist delivered standard CBT-I content in six weekly sessions via website or iOS app. The program was fully automated, and set a lower limit for the time-in-bed recommendation at 6 hours per night. Felder and colleagues compared digital CBT-I to standard care among a sample of pregnant participants with elevated insomnia symptoms (N = 208) [29]. Participants randomized to standard care had no limits placed on receipt of nonstudy treatments, and received a voucher code to Sleepio at study completion. Consistent with hypotheses, participants randomized to digital CBT-I experienced significantly greater improvements from baseline to post-intervention in insomnia severity (ISI; primary outcome) and secondary sleep outcomes (sleep quality as measured by the PSQI; sleep diary-defined sleep efficiency; insomnia caseness as measured by the Sleep Condition Indicator [30]) relative to participants randomized to standard care. Although participants randomized to digital CBT-I did not experience enduring benefits for insomnia severity or sleep quality at 3 and 6 months postpartum, they did evidence significantly higher rates of insomnia remission (53% vs. 35%) at 6 months (but not 3 months) postpartum, relative to standard care [31]. Kalmbach and colleagues found that digital CBT-I also outperformed sleep hygiene education (i.e., 6 weekly emails based on NIH guidance for healthy sleep) in a randomized trial of pregnant people with elevated insomnia symptom severity (N = 91) recruited from a 6-hospital healthcare system in the United States [32]. Evidencing a similar pattern of findings as Felder and colleagues’, participants randomized to digital CBT-I experienced greater improvement in insomnia symptom severity and sleep quality post-intervention, but benefits attenuated in the postpartum period and were not statistically significant at 6 weeks postpartum.

Mackinnon and colleagues investigated telehealth delivery of CBT-I as an option for overcoming practical barriers, including challenges such as transportation, childcare, and work-related constraints. Pregnant participants with elevated insomnia symptom severity (*n* = 62) were randomized to either CBT-I adapted for pregnancy or waitlist treatment as usual (TAU) [33]. Participants assigned to CBT-I received five weekly 60-minute CBT-I individual therapy sessions with a trial therapist under supervision of a licensed psychologist with extensive CBT-I training. In response to the COVID-19 pandemic, intervention delivery shifted from in-person delivery (*n* = 5) to telehealth delivery (*n* = 25). Consistent with other trials, participants randomized to CBT-I had significantly greater improvements in insomnia symptom severity (primary outcome) and sleep quality at post-intervention and six months postpartum, relative to TAU. Multilevel regression model analyses revealed no outcome differences by CBT-I delivery model, suggesting that telehealth may be as effective as in-person therapy for treating insomnia, though further confirmatory work with larger sample sizes is needed. 

In two trials, an Australian research team led by Bei compared CBT-I to experimental sleep interventions targeting different mechanisms. In the first trial, Quin and colleagues evaluated the efficacy of CBT-I adapted for perinatal populations, a responsive bassinet (RB), and sleep hygiene education (SHE) among pregnant participants with elevated insomnia symptom severity (*N* = 127) [24]. CBT-I and SHE were delivered starting in pregnancy (T1, 26–32 weeks gestation) and RB was delivered after birth. Whereas CBT-I was designed to target the cognitive and behavioral perpetuators of maternal sleep disturbance, the responsive bassinet was designed to target infant-related maternal sleep disturbance by reducing infant crying and improving infant sleep with white noise, swaddling, and rocking. CBT-I participants received one 50-minute individual telehealth session with a clinician, 20 intervention emails delivered between baseline and 3 months postpartum, and, if requiring time-in-bed restriction, 3 additional 15-minute phone calls. The authors hypothesized that both CBT-I and RB would outperform SHE; the study was not powered to compare CBT-I to RB. Participants randomized to CBT-I, but not RB, had significant improvements in insomnia severity through one year postpartum (primary outcome) compared to sleep hygiene education.

In the second trial, Verma and colleagues conducted a three-arm randomized controlled trial evaluating six-week CBT-I adapted for perinatal populations, light-dark therapy (LDT), and TAU among individuals 4–12 months postpartum with elevated insomnia symptoms (*N* = 114) [34]. LDT targets circadian influences on sleep by using timed light and dark exposure to realign the biological clock, promote morning alertness, and limit nighttime light exposure. Consistent with hypotheses, both CBT-I and LDT produced significantly greater reductions in insomnia symptoms from baseline to post-intervention relative to TAU (*p*<.001 for both groups); improvements were maintained at 1-month follow-up. Similar patterns were observed for self-reported sleep disturbance. While all other trials reported in this review delivered CBT-I during pregnancy, this trial suggests that CBT-I delivered during the postpartum period is also effective.

### Mindfulness-based Approaches

Our review yielded four trials that investigated mindfulness-based approaches as another method for improving perinatal sleep. Table 2 provides an overview of findings from trials describing mindfulness-based interventions in perinatal populations, including the study population, intervention, comparator(s), primary outcome, timepoints, and results.

**Table 2:** Summary of findings from mindfulness-based trials

Study	Population	Intervention	Comparator(s)	Primary outcome	Timepoints	ISI Results	PSQI Results	Depression Results
Felder et al., 2024	Pregnant; Poor sleep quality	Telehealth MBSR + PS	Treatment as usual (TAU)	Feasibility, acceptability, adherence	Baseline, post-intervention	Not powered for this outcome; Between-group differences in ISI change were non-significant and with small effect size (Cohen’s d = 0.37, favoring MBSR + PS).	Not powered for this outcome; Between-group differences in PSQI change were non-significant and with small effect size (Cohen’s d = 0.28, favoring MBSR + PS).	Not collected.
Kalmbach et al., 2025	Pregnant; Elevated insomnia symptoms	(1)Telehealth PUMAS (2) Telehealth CBT-I	Sleep Hygiene Education (SHE)	Insomnia Severity	Baseline; post-intervention	Greater improvements at post-intervention in PUMAS vs. SHE (-11.04 vs. -4.5, Cohen’s d = 1.87) and CBT-I vs. SHE (-11.2 vs. -4.5, Cohen’s d = 0.84).	Not collected.	Compared to SHE, significantly lower EPDS post-intervention for PUMAS (-3.78, Cohen’s d = 0.83) but not CBT-I.
Wang et al., 2025	Pregnant; Elevated insomnia symptoms	Digital MBI for perinatal insomnia (dMBI-PI)	Treatment as usual (TAU)	Insomnia Severity	Baseline, post-intervention, third trimester, postpartum	Greater reductions from baseline to post-intervention (-5.38 vs. -3.37, Cohen’s d = 0.46) and third trimester (-4.45 vs. -2.43, Cohen’s d = 0.46), but not postpartum (Cohen’s d = 0.12) in dMBI-PI vs. TAU.	Greater improvement from baseline to post-intervention (mean between-group difference − 1.47) and from baseline to the third trimester (mean between-group difference − 1.30) in dMBI-PI vs. TAU.	No significant differences between groups (EPDS) at all timepoints.
Wi et al., 2025	Pregnant; Prior history of depression and currently in remission; Elevated insomnia symptoms	Digital MBI (OPTIMISM)	Education-only Control (EOC)	Sleep Quality	Baseline, post-intervention	Not collected.	Lower post-intervention scores (5.4 vs. 7.6) in digital MBI vs. EOC.	No significant differences between groups (EPDS) post-intervention.

Abbreviations: *CBT-I* Cognitive Behavioral Therapy for Insomnia, *ISI* Insomnia Severity Index, *PSQI* Pittsburgh Sleep Quality Index, *EPDS* Edinburgh Postnatal Depression Scale, *SHE* Sleep Hygiene Education, *TAU* Treatment as Usual, *MBI* Mindfulness-based intervention, *MBSR* Mindfulness Based Stress Reduction, *PS* Perinatal Sleep Classes, *PUMAS* Perinatal understanding of mindful awareness for sleep, *dMBI-PI* Digital Mindfulness-based intervention for perinatal insomnia, *EOC* Education-only Control, *OPTIMISM* Online prenatal trial in mindfulness sleep management

In exit surveys, several participants in Felder and colleagues’ digital CBT-I trial commented that CBT-I did not address the nightly physical symptoms (e.g., discomfort, nocturia) that disrupted sleep [29]. Felder and colleagues theorized that mindfulness-based approaches, which teach skills for coping with pain and discomfort, may be particularly well-suited to address these challenges. For example, mindfulness-based stress reduction (MBSR [35]) teaches nonjudgmental awareness of sensations, thoughts, and emotions and is shown to increase acceptance, reduce rumination, and improve sleep, even in the context of chronic pain, among non-pregnant populations [36, 37]. In a fully remote pilot randomized controlled trial designed to evaluate feasibility and acceptability, pregnant women with poor sleep quality (*N* = 52) were randomized to either TAU or an 8-week program combining standard MBSR with prenatal sleep classes (MBSR + PS) [38]. The PS classes were taught by a clinical psychologist via Zoom and incorporated stimulus control to associate the bed with sleep, time in bed restriction to consolidate sleep, as well as in-the moment tools for coping with sleep disturbances. The MBSR + PS program surpassed all prespecified feasibility (e.g., enrollment, intervention completion, study retention) and acceptability (e.g., satisfaction) benchmarks. Relative to TAU, it showed improvements in intended targets (e.g., self-kindness), and sleep efficiency and restfulness. Together, these preliminary findings show that MBSR + PS is feasible and acceptable, but further work is needed to establish efficacy.

Since then, there have been two adequately powered trials designed to test efficacy of mindfulness-based interventions for perinatal sleep [39, 40]. Kalmbach and colleagues’ cognitive-arousal model provides another theory for why mindfulness may be particularly effective in treating perinatal insomnia. Whereas Felder and colleagues emphasize pregnancy-related physical symptoms as key drivers of sleep disruption, Kalmbach and colleagues identify nocturnal cognitive arousal (e.g., worry, rumination, and perseverative thinking) as a promising, understudied mechanism predicting response to insomnia intervention [41]. Kalmbach and colleagues developed the Perinatal Understanding of Mindful Awareness for Sleep (PUMAS) program, which integrates behavioral sleep strategies (e.g., stimulus control, sleep restriction) within a mindfulness-based therapy framework for insomnia, in six telemedicine sessions delivered 1:1 [39, 41]. In a trial comparing PUMAS, CBT-I, and SHE in sample of pregnant individuals with elevated insomnia symptoms (*N* = 64), PUMAS and CBT-I produced significantly greater reductions from baseline to post-intervention in insomnia severity relative to SHE. PUMAS, but not CBT-I, produced greater reductions in cognitive arousal and depressive symptoms compared to SHE [39]. Thus, these findings suggest that a mindfulness-based intervention specifically targeting nocturnal cognitive arousal among pregnant individuals with insomnia may yield more substantial benefits for depressive symptoms than CBT-I.

In addition to showing promise when delivered live by clinician [38, 39], mindfulness-based interventions for perinatal sleep also appear effective when delivered via self-guided, online platforms. For example, in a single-blind RCT in China, Wang et al. tested a six-week digital mindfulness-based prenatal insomnia program (dMBI-PI) versus standard care in pregnant individuals with elevated insomnia symptoms (*N* = 160) [40]. Relative to standard care, participants randomized to dMBI-PI reported significantly greater reductions in insomnia severity from baseline to post-intervention and third trimester, but not 42 days postpartum. Secondary outcomes (fatigue, mood, and actigraphy metrics) showed no significant between-group differences. Similarly, Wi and colleagues evaluated a six-week online mindfulness program (Online Prenatal Trial in Mindfulness Sleep Management, OPTIMISM [42]) compared to an educational control in a sample of pregnant women with elevated insomnia symptoms (*N* = 45) [43]. Authors concluded that the mindfulness-based intervention was feasible, acceptable, and associated with greater baseline to post-intervention reductions in sleep quality (primary outcome) relative to control. Impacts on secondary sleep and mental health outcomes were mixed.

## Conclusions

Across randomized controlled trials published since 2020, behavioral sleep therapies show largely consistent benefits for insomnia symptoms during pregnancy and postpartum. CBT-I has the strongest evidence base and is shown to improve insomnia severity and sleep quality, whether delivered in person or via telehealth or fully automated digital formats. Findings suggest that the benefits of CBT-I delivered during pregnancy attenuate in the early postpartum period and re-emerge by six months, potentially when nighttime caregiving responsibilities lessen as infant sleep becomes more consolidated. Future research should investigate impacts beyond six months postpartum. CBT-I has been evaluated exclusively in highly controlled efficacy trials with stringent eligibility criteria and behavioral run-ins (e.g., multi-step enrollment process with sleep diaries) to confirm ability to engage with study procedures and maximize retention. Additionally, digital CBT-I trials offered levels of intervention support unavailable in most clinical settings. Accordingly, an important next step for research is to move beyond highly controlled efficacy trials to real world effectiveness trials that: include patients with comorbid sleep disorders, mental health disorders, and high risk pregnancies; rely on prenatal care clinics to identify and refer potentially eligible patients; and eliminate extensive pre-randomization enrollment procedures that limit scalability and generalizability.

Mindfulness-based approaches demonstrate strong feasibility, acceptability, and consistent improvements in subjective sleep, with benefits likely driven by reductions in presleep arousal, worry, physical discomfort, and negative reactivity rather than changes in objective sleep. Most mindfulness-based trials included a short period of follow-up, and more work is needed to evaluate long-term benefits into the postpartum period. Additionally, further research is needed to identify the patient characteristics that should guide clinicians in recommending a mindfulness-based approach instead of CBT-I (e.g., nocturnal cognitive arousal; insomnia symptoms primarily attributed to pregnancy-related physical symptoms).

Across all included trials, samples were relatively homogenous, stressing the need for further investigation among underrepresented racial and ethnic groups. In light of evidence that maternal sleep is associated with offspring outcomes [10–16], future work should evaluate how improving perinatal insomnia affects mother-child interactions, parenting behavior, and child cognition and social-emotional development. Finally, several studies suggested that behavioral sleep therapies delivered during the perinatal period may show promise for reducing or preventing perinatal depression [24, 29, 31, 39]. Of note, two large, adequately powered trials are currently underway to investigate the efficacy of digital CBT-I (https://clinicaltrials.gov/study/NCT05596318) and a mindfulness-based sleep program (https://clinicaltrials.gov/study/NCT06430333) for the prevention of perinatal depression.

## Key References

- Manber R, Bei B, Suh S, et al. Randomized controlled trial of cognitive behavioral therapy for perinatal insomnia: postpartum outcomes. J Clin Sleep Med. 2023;19(8):1411–1419.
  - ○ Face-to-face CBT-I adapted for pregnancy resulted in improved wake after sleep onset and insomnia symptom severity in the postpartum period, relative to active control.
- Felder JN, Epel ES, Neuhaus J, Krystal AD, Prather AA. Randomized controlled trial of digital cognitive behavior therapy for prenatal insomnia symptoms: effects on postpartum insomnia and mental health. Sleep. 2022 Feb 14;45(2):zsab280. 10.1093/sleep/zsab280. PMID: 34850238; PMCID: PMC8842335.
  - ○ Digital CBT-I delivered during pregnancy resulted in improved insomnia severity and lower depressive and anxiety symptoms in the postpartum period, relative to standard care.
- Verma S, Quin N, Astbury L, Wellecke C, Wiley JF, Davey M, Rajaratnam SMW, Bei B (2023). Treating postpartum insomnia: a three arm randomised controlled trial of cognitive behavioural therapy and light dark therapy. Psychological Medicine 53, 5459–5469. 10.1017/S0033291722002616.
  - ○ CBT-I and light dark therapy delivered during the postpartum period resulted in improved insomnia symptom severity, relative to TAU.
- Quin N, Tikotzky L, Astbury L, Spina MA, Fisher J, Stafford L, Wiley JF, Bei B. Preventing postpartum insomnia: findings from a three-arm randomized-controlled trial of cognitive behavioral therapy for insomnia, a responsive bassinet, and sleep hygiene. Sleep. 2024 Aug 14;47(8):zsae106. 10.1093/sleep/zsae106. PMID: 38736364; PMCID: PMC11321850.
  - ○ CBT-I delivered in pregnancy, but not a responsive bassinet used during postpartum, resulted in improved insomnia symptom severity relative to sleep hygiene education.
- Kalmbach, D. A., Ong, J. C., Cheng, P., Reffi, A. N., Swanson, L. M., Hirata, M., Seymour, G. M., Castelan-Cuamatzi, A. S., Jennings, M. B., Pitts, D. S., Roth, A., Roth, T., & Drake, C. L. (2025). A randomized controlled trial of telemedicine CBTI and PUMAS for prenatal insomnia: Reducing nocturnal cognitive arousal is a treatment mechanism for alleviating insomnia and depression during pregnancy. Sleep Medicine, 133, 106570.
  - ○ Telemedicine CBT-I and a mindfulness-based intervention improved prenatal insomnia severity relative to sleep hygiene education, while only PUMAS reduced prenatal depressive symptoms relative to sleep hygiene education.
- Wang, J., Yang, Q., Cui, N., Wu, L., Zhang, X., Sun, Y., Huang, Y., & Cao, F. (2025). Effectiveness and Mechanisms of a Digital Mindfulness-Based Intervention for Subthreshold to Clinical Insomnia Symptoms in Pregnant Women: Randomized Controlled Trial. Journal of medical Internet research, 27, e68084. 10.2196/68084.
  - ○ A digital mindfulness-based intervention for perinatal insomnia improved insomnia severity in pregnant women from baseline through the third trimester relative to treatment as usual.

## Data Availability

No datasets were generated or analysed during the current study.
